# Spatial identification of potential health hazards: a systematic areal search approach

**DOI:** 10.1186/s12942-017-0078-8

**Published:** 2017-02-07

**Authors:** Alina Svechkina, Marina Zusman, Natalya Rybnikova, Boris A. Portnov

**Affiliations:** Department of Natural Resources and Environmental Management, Faculty of Management, University of Haifa, 3498838 Mount Carmel, Haifa, Israel

**Keywords:** Source-oriented models, Receptor-oriented models, Systematic search approach, Disease hotspots, Wind adjustment, Multivariate regression analysis

## Abstract

**Background and aims:**

Large metropolitan areas often exhibit multiple morbidity hotspots. However, the identification of specific health hazards, associated with the observed morbidity patterns, is not always straightforward. In this study, we suggest an empirical approach to the identification of specific health hazards, which have the highest probability of association with the observed morbidity patterns.

**Methods:**

The morbidity effect of a particular health hazard is expected to weaken with distance. To account for this effect, we estimate distance decay gradients for alternative locations and then rank these locations based on the strength of association between the observed morbidity and wind-direction weighted proximities to these locations. To validate this approach, we use both theoretical examples and a case study of the Greater Haifa Metropolitan Area (GHMA) in Israel, which is characterized by multiple health hazards.

**Results:**

In our theoretical examples, the proposed approach helped to identify correctly the predefined locations of health hazards, while in the real-world case study, the main health hazard was identified as a spot in the industrial zone, which hosts several petrochemical facilities.

**Conclusion:**

The proposed approach does not require extensive input information and can be used as a preliminary risk assessment tool in a wide range of environmental settings, helping to identify potential environmental risk factors behind the observed population morbidity patterns.

**Electronic supplementary material:**

The online version of this article (doi:10.1186/s12942-017-0078-8) contains supplementary material, which is available to authorized users.

## Background

Air pollution from motor traffic and industrial facilities is known to be linked to respiratory, cardiovascular and cancer morbidity [1–9]. However, since urban areas are often characterized by multiple sources of air pollution, the identification of specific environmental hazards associated with the observed morbidity patterns is not always straightforward [10–13].

Traditional methods, used to identify the specific sources of air pollution, include the residence time analysis (RTA) and the chemical mass balance (CMB) method [14–22]. The former method is based on measurements of different air pollutants at the receptor sites [15, 18, 20, 23], while the CMB method investigates the chemical composition of air particles, by comparing them with particles emitted from different emission sources [14, 16, 22]. However, the empirical implementation of these methods requires a considerable amount of information on the concentration of specific particles, detailed wind regime assessments and topographic attributes, which are not always available to researchers [14, 24–26].

In this study, we suggest an empirical approach to the identification of specific health hazards, which have the highest probability of being associated with the observed morbidity patterns. The proposed approach does not require extensive input information and can be implemented at a *preliminary* risk assessment stage, using basic geo-statistical tools. The proposed method is based on an expectation that the morbidity effect of a particular health hazard weakens with distance [9, 27–29]. As a result, people living in a close proximity to a morbidity source, are expected to exhibit, *ceteris paribus*, a higher rate of morbidity than those living at a distance from that source [11, 30]. To account for this effect, we estimate distance decay gradients of morbidity for alternative potential “source” locations and then rank these locations based on the strength of association between the observed morbidity patterns and wind-direction weighted proximities to these locations.

## Spatial identification of pollution sources and morbidity hotspots

Empirical implementations of morbidity source assessments can be classified into two groups: *source*-*oriented* approaches and *receptor*-*oriented* methods [14, 15, 20, 23, 24, 26, 31–33]. The first group of methodologies uses data from different pollution sources and then computes the concentrations of different air pollutants in a given point of space, by taking into account local meteorological conditions and topography [32, 34]. By contrast, the second group of methods uses data on air pollution measured at the pollution receptors’ sites and then estimates probable pollution sources, by taking into account the backward wind trajectory and other relevant meteorological conditions (see *inter alia* [14, 20, 35]).

In an early study [15], an identification method of potential emission sources of sulphur dioxide (SO_2_) was developed. The method uses SO_2_ concentrations measured at the receptor site and then calculates a backward trajectory leading to the potential emission source. In a separate study, [36] discuss the results produced by a chemical transport modeling of particulate matter (PM_2.5_), using data available for Northern Italy. According to the proposed method, ambient air pollution is partitioned between road transport, industries and domestic heating.

In several health geography studies, distances from residential locations to pre-identified environmental hazards are commonly used as proxies for unknown (or unidentified) exposures [37–40]. Potential health hazards, to which this exposure assessment method was applied, included highways, industrial sites, nuclear power plants and gas wells.

Thus, in a recent study, McKenzie et al. [6] estimated the health risk associated with areal proximity to natural gas wells in the Garfield County, Colorado. In a separate study, Sermage-Faure et al. [38] investigated the risk of childhood leukemia around nuclear power plants. The total of 32,753 study subjects were subdivided into groups, according to their residential proximity to the existing power plants, and the observed cancers incidence rates across different proximity bands were mutually compared. The results suggested an excess of leukemia in close proximity to nuclear power plants.

Zusman et al. [11] used proximity to an oil storage site, as a proxy for residential exposure to unknown levels of emissions of volatile and semi-volatile organic compounds from the site. As the study revealed, the rates of lung and non-Hodgkin lymphoma (NHL) cancers declined in line with distance from the storage site, especially among the elderly (P < 0.01). A similar methodological approach was used by [30], who investigated the link between NHL morbidity and residence near heavy roads. In the study, the geographic distribution of NHL patients was adjusted by the overall density of population residing in the study area. The analysis indicated a steady decline in the density of NHL patients as a function of distance from main thoroughfare roads.

Although in the above mentioned and other studies (see *inter alia* [6, 41–43]), areal proximities were used for assessing the adverse effects of different health hazards on human morbidity, this method was mostly applied to *pre*-*identified* health risk sources, that is, health hazards found at *known* locations—such as roads, industrial sites, etc.

In the past decades, several geo-statistical tests have been also developed to assess disease clusters around predefined sources of environmental hazards. These tests include Stone’s Maximum Likelihood Ratio Test [44], Tango’s Focused Test [45], Bithell’s Linear Risk Score Test [46], and the Lawson-Waller Score Test [47], also known as the “focused tests”. Although these tests can be used to identify cluster of events around a single or several *pre*-*specified* locations, they cannot be used effectively if the source (or sources) of exposure is unknown, the task which the proposed identification method, based on a systematic areal comparison of alternative risk-source locations controlled for confounders, is designed to achieve.

## Methods

### Identification methodology

Assuming that the rate of morbidity observed in the *i*th point of space (*morb*_*i*_) depends on the distance from the potential source of exposure, *j*, the relationship between *morb*_*i*_ and *dist*_*ji*_ can be expressed by the following linear function, reflecting a monotonic decline in *morb*_*i*_ as a function of *dist*_*ji*_:

1 $$ morb_{i} = b_{0} + b_{1} \cdot dist_{ji} + \varepsilon_{i} . $$

where *b*_*0*_ *, b*_*1*_ are coefficients, $\varepsilon_{i}$ = random error term.

As long as the relationship between *morb*_*i*_ and *dist*_*ji*_ follows (1) and the locations of specific sources of exposure (e.g., roads, industrial facilities, etc.) are a priori known, the calculation of the strength of association between *morb*_*i*_ and *dist*_*ji*_ is technically simple. However, if actual sources of exposure for *morb*_*i*_ for are *unknown*, alternative locations, *j,* can be assessed, one by one, as potential exposure sources. Such alternative locations can then be ranked by their “probability” of being the exposure source (*P*_*ji*_) for *morb*_*i*_ using the coefficient of determination, $R_{ji}^{2}$, between *morb*_*i*_ and *dist*_*ji*_:^1^

2 $$ P_{ji} \to R_{ji}^{2} \left( {morb_{i} , dist_{ji} } \right),\quad \forall  b_{1} \in \left( { - \infty , 0} \right). $$

The interpretation of (2) is relatively simple: values of $R_{ji}^{2}$ close to 1 (when *b*_*1*_ is negative) would indicate a high “probability” that exposure originating from point *j* is associated with morbidity observed in *i*, while values of $R_{ji}^{2}$ close to 1, when *b*_1_ is positive, would indicate a “protective” effect, and values of $R_{ji}^{2}$ close to *zero* will point out at the absence of any significant association between the two variables.

Since the dispersion of air pollutants from a potential risk source is likely to be affected by the wind frequency of from *j* to *i* [48, 49], the pairwise Euclidian distances (*dist*_*ji*_) can be adjusted:

3 $$ \widetilde{dist}_{ji} = T\left( {dist_{ji} \left| {W_{ji} } \right. } \right), $$

where $\widetilde{dist}_{ji}$ = distance between *i* and *j* adjusted by wind frequency (*W*_*ji*_) between the points (measured as e.g., annual or seasonal averages of directional wind frequencies), and $T\left( {dist_{ji} \left| {W_{ji} } \right. } \right)$ is a distance transformation function (e.g., linear, quadratic and exponential transformations can be used; see “Appendix 2”).

To account for the above wind-adjustment effect, (1) can be rewritten as follows:

4 $$ morb_{i} = f\left( {\widetilde{dist}_{ji} } \right). $$

Considering that the association between the observed health effect and proximity to a given health hazard can be confounded by other factors (such as e.g., socio-economic status of the local population, residential densities, ethnicity, etc. [5, 13, 50–52]), the *confound* relationship between the rate of morbidity observed in *i* and $\widetilde{dist}_{ji}$ can be adjusted as follows:

5 $$ morb_{i} = b_{0} + b_{1} \cdot \widetilde{dist}_{ji} + b_{2} \cdot {\mathbf{GEO}} + b_{3} \cdot {\mathbf{SES}} + b_{4} \cdot {\mathbf{POL}} + \varepsilon_{i} , $$

where *b*_*0*_,…, *b*_*4*_ are regression coefficients; GEO = vector of geographical attributes of *i* (e.g., distance to main roads, elevation above the sea level, etc.); SES = vector of socio-economic attributes of *i*, including e.g., socio-economic status and ethnic makeup of the local population; POL = vector of air pollution levels measured at the *i*th point, and $\varepsilon_{i}$ = random error term.

As with (1), the coefficient of determination obtained for (5) can be considered as a measure of probability that morbidity observed in *i* and originated from *j*:

6 $$ P_{ji} \to R_{ji}^{2} \left( {morb_{i} ,\widetilde{dist}_{ji} ,{\mathbf{GEO}},{\mathbf{SES}},{\mathbf{POL}}} \right),\quad \forall  b_{1} < 0. $$

The interpretation of (6) is similar to that of (2): in particular, values of $R_{ji}^{2}$ close to 1 (when *b*_*1*_ is negative) indicate a high “probability” that exposure originating from point *j* is associated with morbidity observed in *i*, while values of $R_{ji}^{2}$ close to 1 (when *b*_*1*_ is positive) would indicate a “protective” effect, and values of $R_{ji}^{2}$ close to *zero* will point out at the absence of any significant association between the variables. The essential difference between (2) and (6) is that the former equation is uncontrolled for potential confounders, while the latter Eq. (6) takes such confounders into account.

### Empirical validation

**Fig. 1:**
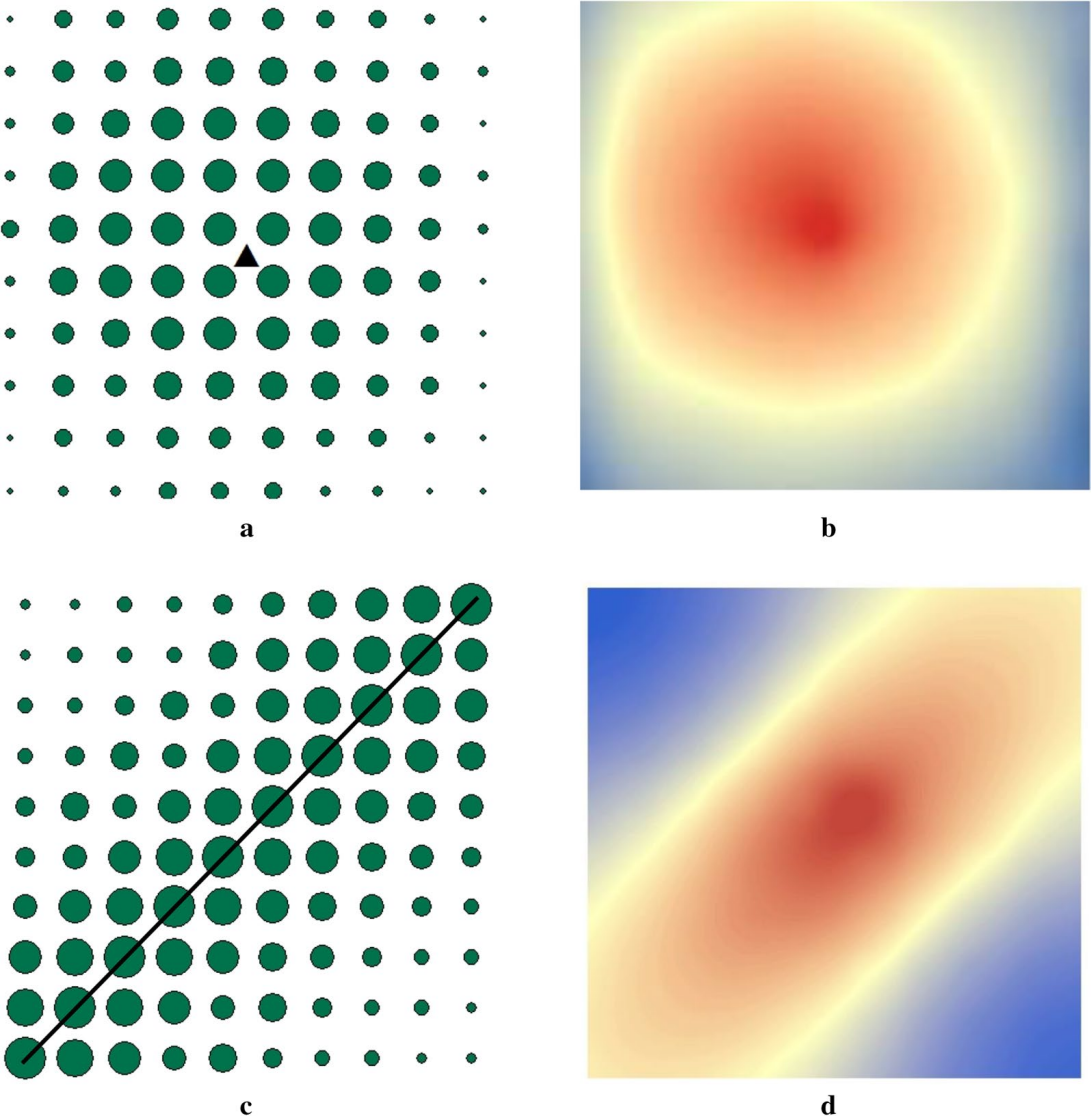
Input morbidity patterns (*left panel*) and risk source estimates (*right panel*). *Notes*: Green dots in the *left panel diagrams* indicate morbidity observations with the size of each dot set proportional to the morbidity prevalence rate observed in a given location; the *triangle* and the *solid line* in the *left panel* diagrams indicate predefined sources of environmental pollution (see text for explanations)

We tested the proposed identification approach in two stages. During the first stage, we designed several theoretical examples in which *loci* of morbidity rates were positioned around pre-defined sources of exposure. In particular, we generated two identical, regularly spaced arrays of 100 “reference” point each, surrounding two predefined sources of exposure—either a point or a line (see Fig. 1; left panel). These arrays of “reference” points served in our tests as both disease observations and points from which potential exposure could have been generated. The rates of morbidity were arbitrarily assigned to each reference point using one simple rule: in line with the expected distance decay relationship, reference points with higher morbidity rates were positioned closer to the pre-defined sources of exposure, while places with lower morbidity rates were positioned farther away from these sources (see Fig. 1; left panel). Then, we estimated bivariate regressions to assess the strength of association between *morb*_*i*_ and *dist*_*ji*_ for each “reference” point (a total of 100 equations, one for each reference point).

We also incorporated a stochastic element into our analysis. In particular, in order to test the sensitivity of the models under varying levels of inputs, we used a random number generator to generate stochastic noise around the input morbidity rates in our “point” and “line” examples (see Fig. 1). Next, we ran 100 regressions for each of the simulated samples. The test did not change the regression results substantially. In particular, in the case of the “point” source (see Fig. 1b), the estimates for the distance variable were as follows: B = −11.17 (95% CI −11.71, −10.63), *t*-stat = −40.84, (95% CI −41.163, −40.76), and for the “line” source (Fig. 1d): B = −5.90 (95% CI −7.98, −3.82), and *t*-stat = −5.47 (95% CI −5.63, −5.36). This confirms that our estimates are essentially robust.

Lastly, we interpolated the *R*_*ji*_^2^ values, to create continuous “probability” surfaces, differentiating between areas with high and low values of the coefficients of determination (see Fig. 1; right panel). To this end, we used the Empirical Bayesian Kriging (EBK) method, a kriging interpolation technique, which differs from classical kriging methods by accounting for the error introduced by estimating the semivariogram model [17, 53]. The EBK parameters were set to the default values used by the ArcGIS^TM^10.x software [54].

**Fig. 2:**
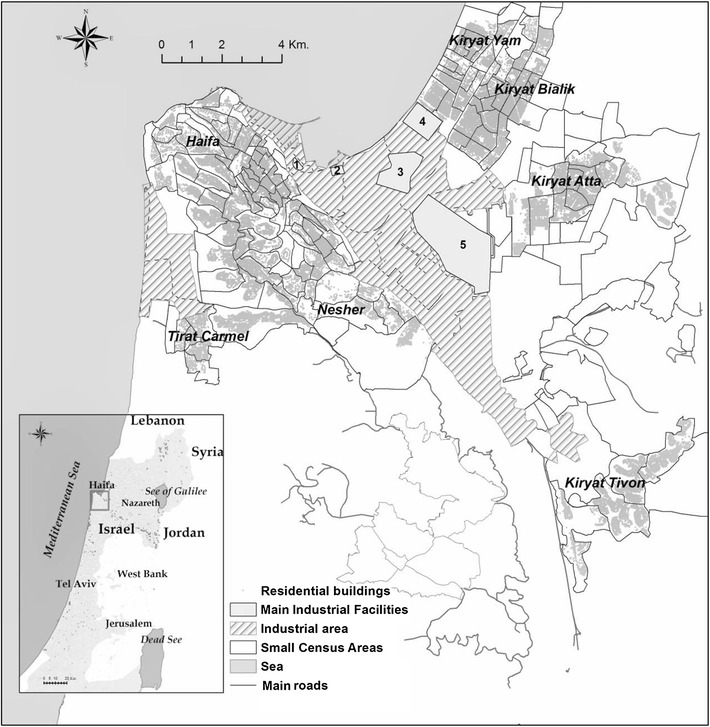
Map of the GHMA study area, showing residential buildings, main industrial facilities (1–5) and thoroughfare roads

At the next step, we applied the proposed identification method to the real world case of the Greater Haifa Metropolitan Area (GHMA) in Israel (Fig. 2), characterized by multiple health hazards. Background information on the study area, its location and geographic attributes is reported in the Additional file 1.

We started our analysis of morbidity patterns in GHMA by geocoding residential addresses of lung and NHL cancer patients, obtained from the Israel National Cancer registry for the year 2012 [55], which are the latest annual records available in the database at the time of the study initiation.^2^ Next, we calculated cancer rates in different areas of the GHMA, using the Double Kernel Density (DKD) tools (see Additional file 2).

In order to convert the obtained continuous DKD surfaces of cancer density into discrete observations, suitable for a multivariate analysis, we generated 1000 randomly distributed “reference” points covering the entire study area (*i* points). Following the analysis procedure suggested in [11], the reference points created thereby were “spatially joined” with DKD surfaces of both types of cancer under study, enabling us to estimate the cancer morbidity rate for each “reference” point. Using the “spatial join” tool in ArcGIS™10.x software [54], we next assigned the values of several variables, either drawn from small census areas (SCAs) data (such as socio-economic status, percent of residents employed in manufacturing, the share of total population over 65yo and neighborhood level smoking rates) or generated from NO_x_ and PM_2.5_ air pollution surfaces, to each reference point.

The air pollution surfaces were interpolated by kriging using annual averages of air pollution obtained from air quality monitoring stations. According to previous studies, cancer latency period can vary substantially, ranging from several years to several decades [57]. To account for this effect, annual averages of NO_x_ and PM_2.5,_ obtained from 20 Air Quality Monitoring Stations (AQMSs) [58], were lagged by 10 years, which is a temporal lag, commonly used in epidemiological studies of cancer [59–61]. That is, cancer DKD rates estimated for the year 2013 were mutually compared with air pollution data for the year 2003 (see Appendix 1).

We considered the above mentioned variables as potential confounders for the observed cancer rates, as commonly done in epidemiological studies of cancer morbidity [5, 13, 51, 52]. Descriptive statistics of the variables used in the analysis are reported in “Appendix 1”.

At the next step, we generated a map (layer) of 1000 evenly distributed points, representing locations of potential environmental hazards (*j* points). For the arrays of *i* and *j*, we next calculated Euclidian distances (*dist*_*ji*_), from each morbidity point (*i*) to each source points (*j*). After these distance pairs were calculated, we introduced them into regression models as potential explanatory variables, in addition to the above mentioned socio-demographic and geographic attributes, considered as controls. To address the issue of multicollinearity, individual *dist*_*ji*_ were introduced into the models separately, one by one, in addition to the constant set of controls, and changes in the coefficient of determinations were traced. The models were estimated separately for two dependent variables—NHL and lung cancer DKD rates.

Because simple Euclidian distances may not be a truly accurate proximity matrix, considering wind frequency and direction, we adjusted these distances by applying a wind frequency transformation discussed in “Appendix 2”. By way of this transformation, we calculated wind weighted distances between each pair of *i* and *j* $\left( {\widetilde{{dist_{ji} }}} \right)$ and then used these wind-adjusted distances in the regression analysis as alternatives to simple Euclidean distances, used during the initial phase of the analysis.

Next, for each morbidity reference point (*i*), we ran multivariate regressions for both types of cancer under study (that is, lung and NHL cancer separately), using the constant set of the above mentioned socio-demographic explanatory and adding one $\widetilde{{dist_{ji} }}$ at a time. For 1000 multivariate regressions obtained for each type of cancer (that is, one regression equation for each *j* point), we used the coefficient of determination $(R_{ji}^{2} )$ to generate the “probability” surface, covering the entire study area and estimating how well the constant set of socio-demographic variables and wind weighted distance from each potential source point *j*, to the disease observation point *i* explain cancer rates observed at *i*’s.

**Fig. 3:**
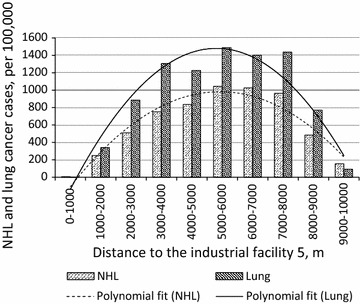
Changes in NHL and lung cancer incidence rates (per 100,000) as a function of distance from industrial facility 5 (see Fig. 2)

In the initial stage of the analysis, $\widetilde{{dist_{ji} }}$ were introduced by their linear terms. However, as our analysis revealed, the relationship between the observed cancer morbidity and industrial proximities was best captured by a non-linear (parabolic) function (see Fig. 3), apparently due to the fact that plumes of air pollution from tall industrial smokestacks land at some distances from the emission sources. To take this non-linear effect into account, we introduced a quadratic term of $\widetilde{{dist_{ji} }}$ into the models, in addition to its linear term, and repeated the analysis. To estimate parameters in Eq. (5) multivariate regression models, incorporating linear and non-linear terms, were used. In the following discussion, *only* non-linear models, providing better fits and generality compared to ordinary linear models, are reported.

The probability surfaces were generated using the EBK interpolation technique in the ArcGIS™10.x Software [54], while the multivariate regression analysis was performed using the SPSSv.23™ software [62]. The probability level of less than 0.01 (<1%) was set as the accepted statistical significance level.

## Results

### Theoretical examples

Figure 1 features morbidity rates, marked by dots surrounding two *pre*-*defined* sources of exposure—a triangle (Fig. 1a) and a line (Fig. 1c). As mentioned previously, in these diagrams, dots, marking morbidity observations, are sized proportionally to the predefined morbidity rates: the higher the morbidity rate: the bigger the dot that marks it. In line with the expected distance decay relationship, larger dots are positioned closer to the pre-defined sources of exposure, while smaller dots are placed farther away from these sources (see Fig. 1a, c). Concurrently, maps in the right panel (Fig. 1b, d) feature morbidity source estimates, calculated using the estimation approach described in “Empirical validation” section. As Fig. 1b, d show, the spots of high probability of being the source of exposure, marked by orange and red colours in the right panel, correspond, fairly accurately, to the actual locations of the pre-defined health hazards (Fig. 1a, c).

### GHMA study

**Fig. 4:**
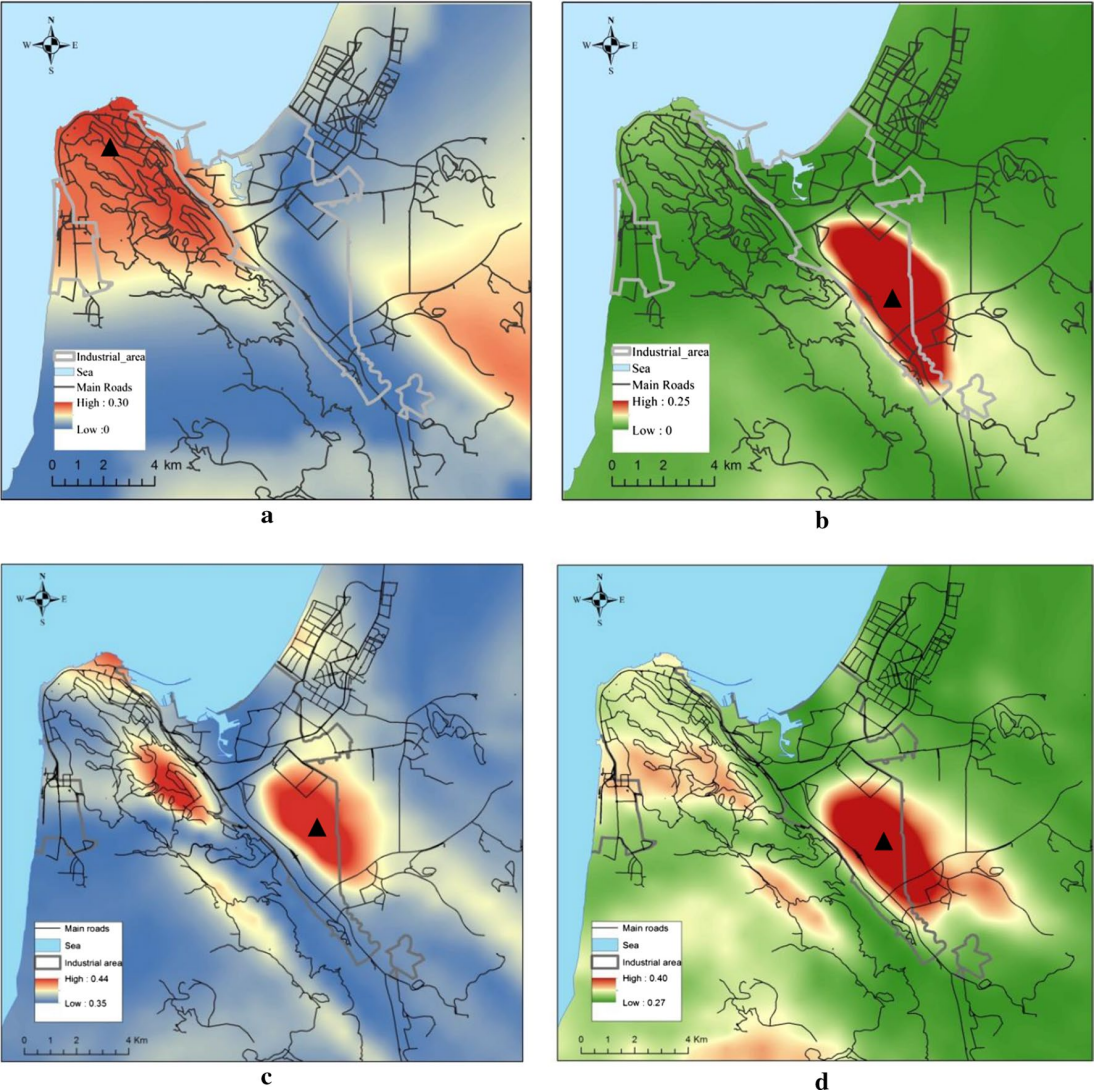
Risk source assessment for lung cancer (*left panel*) and NHL cancer (*right panel*) by uncontrolled (**a**, **b**) and controlled regressions (**c**, **d**). *Note*: *Black triangles mark* the points, distances to which are used in the regression models reported in Tables 1 and 2

Figure 4a, b shows raster surfaces based on the determination coefficients ($R_{ji}^{2}$), obtained from *bivariate* regression models, estimated separately for lung (Fig. 4a) and NHL cancers (Fig. 4b). Concurrently, Fig. 4c, d shows source identification surfaces based on the determination coefficients obtained from *multivariate* regression models. The best performing regression models (both controlled and uncontrolled), are reported in Tables 1 and 2.^3^

**Table 1:** The association between double kernel density (DKD) of lung and NHL morbidity rates (cases per 100,000 residents) and distance to the revealed exposure sources (Method—*bivariate* regression, distance variables—linear and quadratic wind-adjusted distance terms)^c^

Variables	Model 1	Model 2
	B^a^ and (t^b^)	B^a^ and (t^b^)
A. Lung cancer		
(Constant)	13.935 (58.947*)	1.131 (7.965*)
Distance	−5.500E−0.40 (−19.350*)	0.002 (4.364*)
Distance^2^	–	−1.115E−07 (−4.152*)
No. of reference points	1000	1000
${\text{R}}^{2}$	0.286	0.301
${\text{R}}_{\text{adjusted}}^{2}$	0.285	0.299
F	374.419*	133.819*
B. NHL cancer		
(Constant)	4.656 (17.237*)	−3.697 (−5.219*)
Distance	3.380E−04 (8.409*)	0.003 (13.916*)
Distance^2^	–	−2.189E−07 (−12.791*)
No. of reference points	1000	1000
${\text{R}}^{2}$	0.070	0.205
${\text{R}}_{\text{adjusted}}^{2}$	0.069	0.204
F	70.714*	120.722*

Model 1: Bivariate linear model

Model 2: Bivariate quadratic model

* indicates a 0.01 two-tailed significance level

^a^Regression coefficient

^b^ *t*-statistics in the parentheses

^c^The models reported in the table are estimated for the distances to the “best performing” source locations, marked by small triangles in Fig. 4, that is, source locations distances to which help to improve the models’ fits most significantly (see text for explanations)

**Table 2:** The association between double kernel density (DKD) of lung and NHL morbidity cancer rates (cases per 100,000) and distance to the revealed exposure sources (Method—*multivariate* regression, distance variables—linear and quadratic wind-adjusted distance terms)^c^

Variables	Model 3^d^	Model 4^d^
	B^a^ and (t^b^)	B^a^ and (t^b^)
A. Lung cancer		
(Constant)	6.661 (2.591*)	−12.629 (−3.959*)
Distance	−5.159E−04 (−7.470*)	0.003 (8.235*)
Distance^2^	–	−2.620E−07 (−8.159*)
N of reference points	1000	1000
${\text{R}}^{2}$	0.393	0.458
${\text{R}}_{\text{adjusted}}^{2}$	0.386	0.450
ΔR^2^	–	0.065
F change^e^	–	36.658*
B. NHL cancer		
(Constant)	9.119 (5.231*)	−9.144 (−4.388*)
Distance	−2.862E−04 (−5.991*)	0.003 (13.359*)
Distance^2^	–	−2.415E−07 (−12.791*)
N of reference points	1000	1000
${\text{R}}_{{}}^{2}$	0.242	0.369
${\text{R}}_{\text{adjusted}}^{2}$	0.234	0.361
ΔR^2^	–	0.127
F change^e^	–	92.855*

Model 3: Multivariate linear model

Model 4: Multivariate quadratic model

^a^Regression coefficient

^b^ *t*-statistics in the parentheses

^c^The models reported in the table are estimated for the distances to the “best performing” source locations, marked by small triangles in Fig. 4, that is, source locations distances to which help to improve the models’ fits most significantly (see text for explanations)

^d^The models are controlled for distance to the nearest main road (m), elevation above the sea level (m), percent of Jewish population in the SCA, SCA Socio-economic status, distance to the sea (m), manufacturing employment (% of total population of SCA), NO_x_ (ppb), PM _2.5_ (ppb), total population over 65 (%),smoking rate in the SCA (%) and distance to the nearest main road (m)

^e^F-test of R^2^-change compared to model without hazard source distances (i.e., Models 3A or 3B, respectively)

Figure 4 has similar coloring such as that used in the theoretical examples, discussed in “Theoretical examples” section and shown in Fig. 1. In particular, warm-coloured pixels in these diagrams correspond to the highest improvements in the models’ determination coefficients, observed by adding wind-adjusted distances from these pixels to the models, containing a constant “pre-set” of socio-demographic variables, discussed in the “Empirical validation” section. Concurrently, blue and green colours in these maps mark pixels adding proximity to which result in relatively small changes in the models’ determination coefficients.

As Fig. 4 shows, there are two most probable *loci* associated with the observed morbidity—the central business district saturated with traffic routes located in the north-eastern part of the study area (for lung cancer cases) and a spot located in the central part of the study area (for both cancer cases under the study) (see Figs. 1, 4a, b). Adding proximities to these spots results in increases in the models’ determination coefficients by up to 14–29% in bivariate models and by up to 7–13% in multivariate models, depending on the cancer type under analysis (see Tables 1, 2).

Several interaction effects were also tested. Among them, two effects (i.e., the side of the Carmel mountain vs. elevation above the sea level and the side of the Carmel mountain vs. distance to the identified hotspot), were found to be statistically significant. Regression models incorporating these interaction effects are reported in Table 3.

**Table 3:** The association between double kernel density (DKD) of lung and NHL morbidity rates (cases per 100,000 residents) and distance to the revealed exposure sources (Method—*multivariate* regression, distance variables—quadratic wind-adjusted distance terms; interaction terms added)^c^

Variables	Model 5	Model 6	Model 7
	B^a^ and (t^b^)	B^a^ and (t^b^)	B^a^ and (t^b^)
A. Lung cancer			
(Constant)	−15.663 (−7.937*)	−15.125 (−4.832*)	−15.791 (−4.948*)
Distance	0.004 (8.591*)	0.004 (9.314*)	0.004 (8.109*)
Distance^2^	−2.689E−07 (−8.258*)	−2.945E−07 (−9.067*)	−2.715E−07 (−7.587*)
No. of reference points	1000	1000	1000
${\text{R}}^{2}$	0.478	0.480	0.480
${\text{R}}_{\text{adjusted}}^{2}$	0.470	0.471	0.472
F	56.308*	56.582*	56.790*
B. NHL cancer			
(Constant)	−9.890 (−4.709*)	−9.233 (−4.402*)	−10.001 (−4736*)
Distance	0.003 (13.563*)	0.003 (13.119*)	0.003 (12.436*)
Distance^2^	−2.438E−07 (−12.930*)	−2.457 (−11.995*)	−2.486E−07 (−11.079*)
No. of reference points	1000	1000	1000
${\text{R}}^{2}$	0.373	0.374	0.374
${\text{R}}_{\text{adjusted}}^{2}$	0.364	0.364	0.364
F	39.311*	39.288*	36.704*

See comments to Table 2

Model 5: Multivariate quadratic model with the Side of Mountain Carmel vs. elevation above the sea level interaction term

Model 6: Multivariate quadratic model with the Side of Mountain Carmel vs. Distance to the identified hotspot interaction term

Model 7: Multivariate quadratic model with both interaction terms added

## Discussion

Empirical studies use several methods for the spatial identification of potential health hazards. Such methods are mostly based on the measurements of air pollutants at the receptor sites, followed by a comparison of the results of such measurements with the chemical composition of particles emitted from different emission sources [14, 15, 20, 23, 24, 26, 31, 32, 35]. However, the empirical implementation of these methods requires a considerable amount of information on the concentration of specific particles, detailed wind regime assessments and topographic attributes, which are not always available to researchers [14, 20, 26].

As an alternative approach, proximities of various health hazards, such as roadways, industrial sites, nuclear power plants and gas wells, are commonly used in epidemiological and health geography studies as *proxies* for unknown exposures (see *inter alia* [11, 27, 28, 30].

In the present study, we extend this *distance gradient* method to the spatial identification of a priori *unidentified* hazards. The underlying assumption behind the proposed identification approach is that people living in a close proximity to a morbidity source, tend to exhibit, *ceteris paribus*, a higher rate of morbidity than those living at a distance from that source [11, 30]. To account for this effect, we estimated distance decay gradients of morbidity for alternative potential "risk source" locations and then ranked these locations based on the strength of association between the observed morbidity patterns and wind-direction weighted proximities to these locations.

In empirical studies, several measures are commonly used to estimate the improvement of regression models attributed to changes in the predictors’ set. Such measures include the log-likelihood criterion, the Akaike information criterion (AIC), the Bayesian information criterion (BIC), the Schwarz criterion (SBC), Mallow’s C_p_ statistic, and several others. These criteria monitor changes in the regression residuals and thus help to select the combination of explanatory variables and the functional form of the model best fitted to the data under analysis [63]. In this study, we used R^2^, a commonly used measure of model fit, also known as the coefficient of determination. Our preference for this measure was motivated by the fact that this measure does not depend on the order of variables, has a specific interval of change (0; 1); it also does not depend on the functional form of the regression equation used [64]. Using this measure and applying it to the constant set of control variables, we monitored changes in the regression fit attributed to changes in wind adjusted distances to alternative hazard locations, which were introduced into the models one by one. Since the set of control variables used in the study included main factors known to affect cancer incidence rates in urban areas [51, 61, 65, 66], we did not consider it feasible to alter this predetermined set of controls. In other words, according to the proposed identification approach, the coefficient of determination, R^2^, was considered a likelihood criterion, using which we compared several combinations of input parameters. These combinations included the constant set of confounders and a number of vectors of wind-weighted distances between alternative potential health risk sources and morbidity observations.

In several theoretical examples we designed, the proposed approach helped to identify correctly the predefined locations of health hazards, while in a real-world case study, the main health hazard were identified as a spot in the industrial zone, which hosts petrochemical facilities, and a major transportation hub in the central business district of the city. According to previous studies (see *inter alia*, [11, 38, 67]), petrochemical industries are known to be associated with evaluated cancer morbidity in surrounding residential areas. In a separate study, [67] investigated morbidity near nuclear power plants and found it to be linked to childhood cancer.

The results of the present study also correspond to the findings of other studies which revealed geographic concentrations of cancer morbidity near heavy roads [30, 40, 41, 68], and in proximity to industrial areas [11, 38]. Thus, [69] identified the link between traffic-related pollution and respiratory morbidity, measured by lung function impairment.

Several limitations of our study need to be mentioned. First and foremost, the present study is an ecological analysis, in which explanatory variables are measured at the group level or as distance gradients, and not estimated for individuals. Therefore, we cannot attribute causality in the relationships we observed. However, the strength of population-level studies is that they represent large population groups and reflect varying levels of exposure. The purpose of such studies is not to prove the relationships but rather to generate hypotheses which can further be examined using individual level data [70].

## Conclusions

This paper contributes to the existing body of literature by extending the traditional distance gradient method (DGM) to the identification of potential health hazards, which geographic location is a priori *unknown*. The results of the study demonstrate the utility of the proposed method for epidemiological studies which goal is to identify potential sources of exposure to which the observed morbidity is related. We also consider it important that the proposed approach does not require extensive input information and can be used as a preliminary risk assessment tool, helping to identify potential environmental risk factors behind the observed population morbidity patterns.

The proposed approach can be used by researches worldwide in cases in which specific sources of locally elevated morbidity are unclear or cannot be identified by traditional methods. For instance, the proposed method can be used in empirical studies in which available epidemiological data can help to map the existing morbidity patterns, and then to identify potential sources of exposure to which the observed morbidity patterns are related. However, future studies will be needed to extend the theoretical justification of the proposed approach, and to determine its applicability to other urban areas and to other health outcomes.

## Appendix 1

See Table 4.

**Table 4:** Descriptive statistics of the variables used in the multivariate regressions

Variables	Minimum	Maximum	Mean	SD
DKD of NHL cancer cases (per 100,000)	0.00	18.54	6.78	2.49
DKD of Lung cancer cases (per 100,000)	0.00	27.22	10.08	4.04
Average distance to main industrial facilities (m)	755.57	9996.96	5402.55	2095.05
Distance to the nearest main road (m)	0.54	1217.84	163.71	182.13
Distance to the seashore (m)	2.38	14,302.92	4397.48	3872.51
Manufacturing employment (% of total population of the SCA)	0.00	29.30	14.68	6.20
Percent of Jewish population in the SCA	0.00	100.00	91.05	20.54
SCA socio-economic status (Index)	−1.62	2.88	0.44	1.08
NO_x_ in 2003 (IDW interpolation, ppb)	7.68	133.12	27.97	15.00
PM_2.5_ in 2003 (IDW interpolation, ppb)	17.20	27.80	20.11	1.25
Total population over 65 (%)	0.00	0.39	0.17	0.06
Smoking rate in the SCA in 2003 (%)	15.07	41.78	18.87	3.49
Elevation above the sea level (m)	0.00	440.00	110.49	124.83

## Appendix 2: Wind weighted transformation of distances

Adjustments for wind frequency and directions were implemented in a number of studies dealing with the measurements of directional concentrations of urban air pollutants (see *inter alia*, [48, 49, 71, 72]). The empirical literature reports several approaches to distance transformation, based on the seasonal analysis of data [48] or on the amount of precipitation, solar radiation, maximum and minimum temperatures [49] and average wind speed [71].

For the purpose of wind adjustment, we used the wind frequency rose of the study area, plotted at a one-degree angular resolution and showing the average annual distribution of wind frequencies for each one-degree angle. Since the probability of wind from point *j* to point *i* ($w_{ji}$) was assumed to be random, we used the probability density function (*PDF*) instead of a fixed distribution function. PDF describes the relative likelihood for the random variable, e.g., wind frequency to take on a given value (*w*_*ji*_):

7 $$ \begin{aligned} & PDF_{w} \left( {w_{ji} , \lambda_{j} } \right) = \lambda_{j} \cdot e^{{ - \lambda_{j} \cdot W_{ji} }} ,\quad \forall  W_{ji } \in \left( {0,1} \right), \\   & \lambda_{j} = \frac{1}{{\overline{{w_{ji} }} }},\;{\text{for}}\;\overline{{w_{j} }} = \frac{1}{n}\mathop \sum \limits_{i = 1}^{n} w_{ji} . \\ \end{aligned} $$

where $w_{ji}$ is annual wind frequency from point *j* to point *i*, $\overline{{w_{j} }}$ - average annual wind frequency from point *j, n* - is a number of *i* points, $\lambda_{j}$ > 0 is the parameter of the distribution, also known as the *rate* parameter.

Next, distances between *j* and *i* (*dist*_*ji*_) were adjusted for wind frequency and direction as follows:

8 $$ \widetilde{{dist_{ji} }} = T\left( {dist_{ji} \left| {W_{ji} } \right. } \right) = dist_{ji} \cdot PDF\left( {W_{ji} , \lambda_{\text{j}} } \right). $$

According to this transformation, distances between point *j* and *i* with frequent winds are reduced, while distances between points with infrequent winds remain unchanged.

## Appendix 3 Description of mathematical logic symbols used in the manuscript

$\forall$—symbol indicates “for all”, “for any”, “for each”;

$\to$—symbol indicates domain and codomain of a function;

$\in$—symbol indicates affiliation to any set of elements.

## Additional files

**Additional file 1.** Study area.

**Additional file 2.** Double kernel density (DKD) estimates.

## Abbreviations

CMB: chemical mass balance
DGM: distance gradient method
DKD: double Kernel density method
EBK: empirical Bayesian Kriging
GHMA: greater Haifa metropolitan area
KD: Kernel density
NHL: non-Hodgkin lymphoma cancer
PDF: probability density function
RTA: residence time analysis
SCA: small census area

## Footnotes

1 For explanation of mathematical symbols used in the paper, see “Appendix 3”.

2 Geocoding is a process of converting street addresses into geographic (X, Y) coordinates suitable for mapping [56].

3 The models reported in Tables 1 and 2 feature distances to the pixels adding which resulted in the largest changes in the models’ determination coefficients. Such pixels are marked by deep red colors in Fig. 4.
