# Supplementary material for: Spatial identification of potential health hazards: a systematic areal search approach
Source: Int J Health Geogr. 2017 Feb 7;16:5. doi: 10.1186/s12942-017-0078-8 (PMC5297159; doi:10.1186/s12942-017-0078-8)
Supplement: Supplementary file 1 — Additional file 1. Study area. [file 12942_2017_78_MOESM1_ESM.doc]

**Additional file 1:** *Study area*

Greater Haifa Metropolitan Area (GHMA), which forms our study region, consists of the City of Haifa and its suburbs – Krayot, Tirat Carmel, Nesher and Kiryat Tivon, – and is located on the [Mediterranean coast](https://en.wikipedia.org/wiki/Israeli_coastal_plain) (see Fig. 2). The GHMA is the third largest metropolitan area in Israel (after Tel Aviv and Jerusalem), and has the total population of about 600,000 residents, 81% of which are Jews, 11% are Arabs and 8% are others [75].

GHMA is located on the slopes of the Carmel Mountain and is a major center of heavy industry, including pharmaceutical and chemical processing plants, [oil refineries](http://www.haaretz.com/life/nature-environment/.premium-1.608340) and a small power plant. A sea port and an airport are also located in the city proper. Because of industrial activity and heavy traffic, large concentrations of emissions are released to the air, potentially linked to elevated morbidity. In 2015, the Israel Cancer Registry reported that cancer rates in the Haifa region are about 15 percent higher than the national average [56]. The most frequent types of cancer in GHMA are lung cancer (16% of excess rate in men and 25% in women), bladder cancer (8%), and Non-Hodgkin's lymphoma (19% higher than the national average among men and 18% – among women).
