# Supplementary material for: Spatial identification of potential health hazards: a systematic areal search approach
Source: Int J Health Geogr. 2017 Feb 7;16:5. doi: 10.1186/s12942-017-0078-8 (PMC5297159; doi:10.1186/s12942-017-0078-8)
Supplement: Supplementary file 2 — Additional file 2. Double kernel density (DKD) estimates. [file 12942_2017_78_MOESM2_ESM.doc]

**Additional file 2**: *Double kernel density (DKD) estimates*

According to the kernel density (KD) approach, the density of the observed events is calculated for each geographic location, by counting the number of the events of interest with a predefined search radius, also known as the *kernel bandwidth* [76, 77, 78].

In several recent studies [11, 79, 80], a refinement of the KD method, termed the Double Kernel Density (DKD) technique, was proposed. The DKD technique is based on the transformation of continuous KD surfaces into discrete observations, suitable for multivariate analysis. In these empirical studies, kernel smoothing was used to analyze the concentration of lung cancer cases [80], breast cancer cases [79] and lung and NHL cancers [11], and to associate the observed morbidity with underlying environmental causes, such as air pollution, nighttime artificial illumination or residential proximity to petroleum storage tanks. According to these studies, the DKD technique helps to provide better assessment results than traditional zonal approaches, based on aerial aggregation of original observations into predefined areal units, such as neighborhoods or small census areas, especially if the number of geographic units available for the analysis is small.

Given a point-based dataset, composed of discrete geographically referenced observations, KD calculation bases on a non-linear kernel function
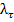
 estimated as follows [82]:


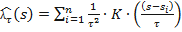
. (AF2.1)

Where *s* is point for which KD is calculated; *s_1_,..., s_n_*, are the vector locations of the *n* observed point events around point *s*; *k( )* represents the kernel weighting function, and *τ* > 0, is the bandwidth parameter, with the value of *τ* chosen (Silverman, 1986).

The result is a raster grid with a calculated kernel density estimates for each point *s*. While the kernel *bandwidth* - the distance from the target location, defines the degree of smoothness of density surface (see Fig. AF2.1).

Figure AF2.1: KD estimation of the point pattern

After a KD surface is calculated, *normalization* is the next step. This step is required to assure that the concentration of patients near e.g., a pollution source is not solely due to high population densities around it. For performing a normalization, the observed density of cancer patients in each point of space divided by the total number of people living in the same location [77, 79]. As a result DKD is estimated as a rate, that is, as a number of disease cases per e.g., 1,000 or 10,000 residents [81].
